# Successful Bronchoscopic Cryorecanalization in a Case of Endobronchial Lipoma

**DOI:** 10.1155/2011/845686

**Published:** 2011-06-06

**Authors:** B. Lamprecht, G. Hutarew, P. Porsch, B. Wegleitner, M. Studnicka

**Affiliations:** ^1^Department of Pulmonary Medicine, University Hospital Salzburg, Müllner Hauptstraße 48, 5020 Salzburg, Austria; ^2^Institute of Histopathology, University Hospital Salzburg, 5020 Salzburg, Austria

## Abstract

Endobronchial lipomas are rare benign tumors; less than 150 cases have been reported so far. Bronchial occlusion usually leads to a misdiagnosis of asthma/COPD or malignancy. We report the case of a 67-year-old man with a history of heavy smoking (100 pack years), dyspnea on exertion, cough, and malaise who was treated for pneumonia for three weeks. Due to nonresolving atelectasis of the superior segment of the right lower lobe, a malignant endobronchial tumor was suspected. Rigid bronchoscopy with cryorecanalization led to both the definite histopathological diagnosis of endobronchial lipoma and the reopening of an endoluminal airway obstruction during one procedure.

## 1. Introduction

Endobronchial lipomas are rare benign tumors; less than 150 cases have been reported so far. Bronchial occlusion usually leads to a misdiagnosis of asthma/COPD [1] or malignancy [2, 3]. On the one hand, many patients with endobronchial lipoma undergo radical procedures such as lobectomy and pneumonectomy [4]. On the other hand, late diagnosis can lead to irreversible pulmonary damage. However, the majority of endobronchial lipomas are located in the first three subdivisions of the tracheobronchial tree, and thus, they are potentially accessible to diagnostic and therapeutic endoscopic techniques.

We report the case of a 67-year-old man who presented with nonresolving pneumonia and atelectasis of the superior segment of the right lower lobe. He was diagnosed with endobronchial lipoma and successfully treated by interventional bronchoscopy.

## 2. Case Presentation

A 67-year-old man with a history of heavy smoking (100 pack-years), dyspnea on exertion, cough, and malaise was treated for pneumonia with amoxicillin-clavulanic acid for three weeks. Due to nonresolving atelectasis of the superior segment of the right lower lobe, a malignant endobronchial tumor was suspected. The patient was referred to our department for further diagnostic workup.

Clinical examination revealed an obese man, with slight dullness to percussion and decreased breathing sounds at the right lower chest. Blood tests showed a C-reactive protein (CRP) of 2.3 mg/dL (<0.6) without leucocytosis. 

**Figure 1:**
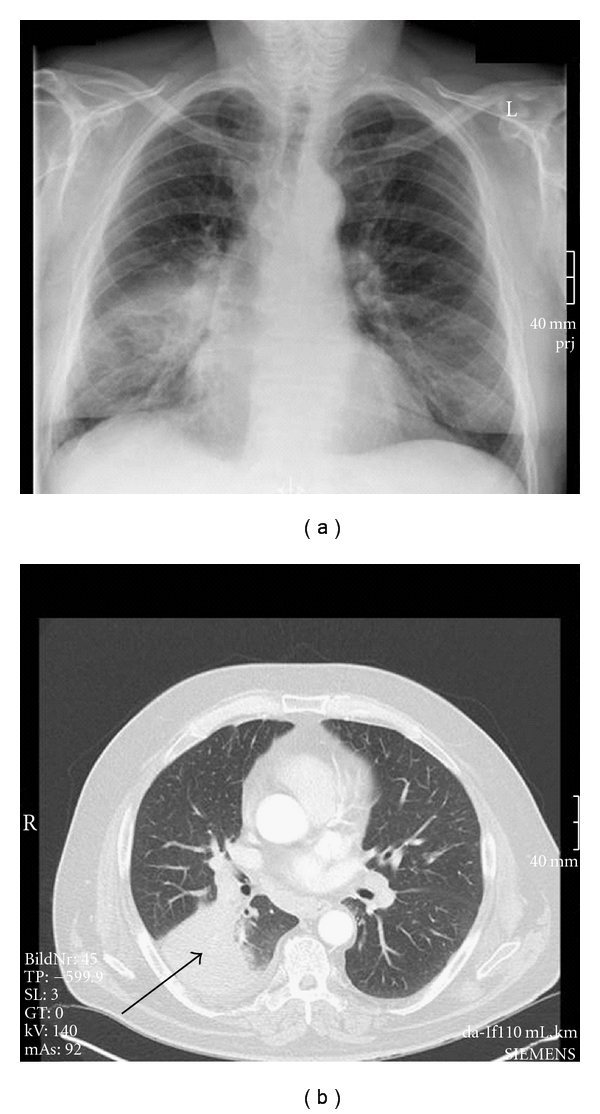
Chest radiograph and chest CT scan before bronchoscopic resection. Arrow indicating atelectasis of the superior segment of the right lower lobe.

On chest radiograph and chest-computed tomography (CT) scan, atelectasis of the superior segment of the right lower lobe was present (see Figure 1). A subsequent PET-CT scan suggested an inflammatory process; however, the presence of a malignant lesion could not be ruled out definitely.

Bronchoscopy was performed in general anesthesia, and a rigid bronchoscope was used for intubation. Endoscopic inspection with the flexible video bronchoscope revealed a total obstruction of the superior segmental bronchi of the right lower lobe by a yellowish round mass. 

**Figure 2:**
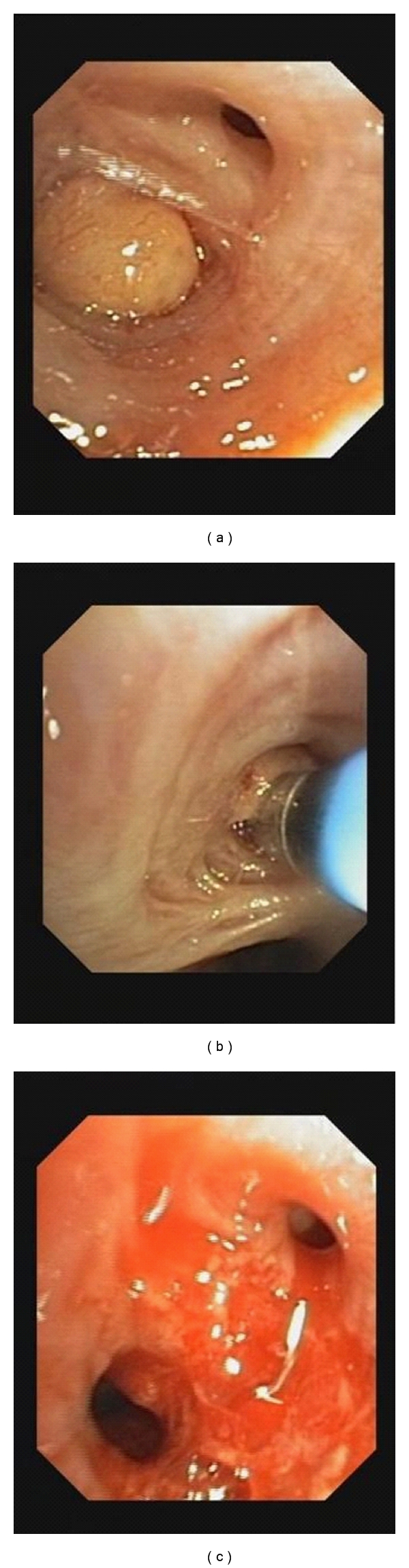
Endobronchial lipoma and successful bronchoscopic cryorecanalization ((a): bronchial occlusion due to endobronchial lipoma; (b): recanalization using the Cryoprobe; (c): successful reopening of the endoluminal airway obstruction).

**Figure 3:**
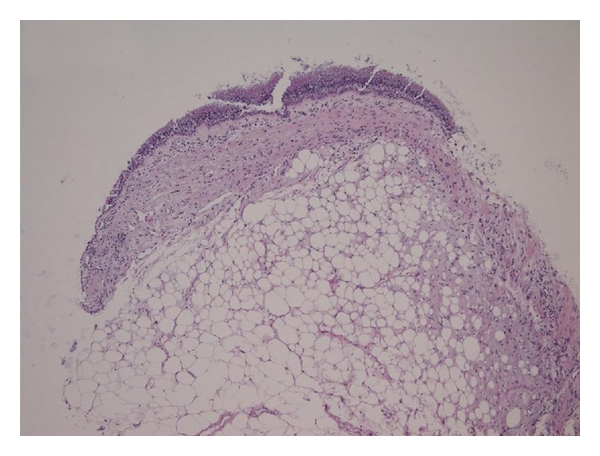
Proliferation of mature adipose tissue with mucoid changes covered by regular bronchial mucosa. Stained with hematoxylin and eosin, 100x magnification.

A flexible cryoprobe was passed through the video bronchoscope for cryorecanalization. Large areas of the tumor were frozen and removed from the bronchial wall. This led to the reopening of the occluded airway see Figure 2. Rapid on-site cytopathologic examination (ROSE) did not suggest a malignant lesion. Histopathological examination of the biopsies confirmed a proliferation of benign adipose tissue covered by bronchial mucosa with normal respiratory epithelium. There was no evidence of malignancy, and thus, a diagnosis of endobronchial lipoma was established; see Figure 3.

**Figure 4:**
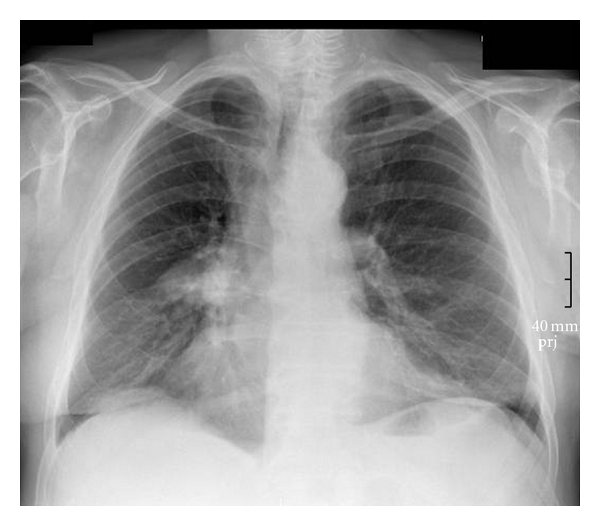
Chest radiograph one month after bronchoscopic resection.

**Figure 5:**
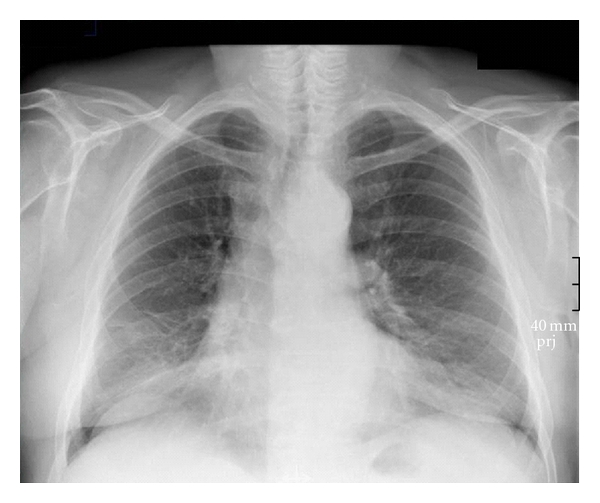
Chest radiograph seven months after bronchoscopic resection.

Bronchoscopy and cryorecanalization allowed diagnosis and treatment of this benign condition during one procedure. The patient recovered, and atelectasis was on the decrease when reviewed one month after the intervention, see Figure 4. Seven months after bronchoscopic intervention; the patient was still free of complaints, and atelectasis had been resolved; see Figure 5.

## 3. Discussion

Endobronchial lipomas are rare and benign tumors without excess risk of malignant potential [5]. However, delay in diagnosis often results in irreversible damage and radical surgical procedures [4, 5]. 

With regard to the diagnostic workup, it has been shown that biopsies obtained by flexible bronchoscopy are often (74%) nondiagnostic [6]. However, tissue obtained during rigid bronchoscopy is almost always diagnostic [6]. Therefore, rigid bronchoscopy under general anesthesia has been recommended for diagnosis and treatment of this benign condition [6]. The low diagnostic yield of flexible bronchoscopy can be explained by the fact that the histopathological feature is located in the submucosa and covered by normal epithelium. However, using the cryoprobe large pieces of the tumor can be extracted, and this can help to overcome the limitation of low diagnostic yield. In our case, all specimens obtained by the cryoprobe were diagnostic. With this results in mind, one could reasonably argue that cryoextraction with a flexible cryo probe allows to pass on rigid bronchoscopy and general anesthesia.

In general, endobronchial resection has been shown to be effective and safe [6]. The endoscopic techniques usually include mechanical debulking, laser [7], electrocautery [8] and cryotherapy [9, 10]. All these techniques are acceptable, and the choice often depends on the physicians preference and local resources.

In the case we report, cryorecanalization of the endobronchial lipoma led to both the definite histopathological diagnosis and the reopening of an endoluminal airway obstruction during one procedure.

It has been concluded that bronchoscopic treatment should be the method of choice as it helps to preserve lung parenchyma. However, surgical resection will be required if there is permanent distal damage or any feature suggesting a possible malignant process [5]. 

Clinicians need to be aware of this rare and benign condition mimicking malignancy, asthma, or COPD. Early diagnosis and endoscopic resection help to prevent irreversible distal lung damage.

##  Conflict of Interests

The authors declare that there is no conflict of interest.
